# Snoo and Dpp Act as Spatial and Temporal Regulators Respectively of Adult Progenitor Cells in the *Drosophila* Trachea

**DOI:** 10.1371/journal.pgen.1005909

**Published:** 2016-03-04

**Authors:** Nareg J.-V. Djabrayan, Jordi Casanova

**Affiliations:** 1 Institut de Biologia Molecular de Barcelona (CSIC), Barcelona, Catalonia, Spain; 2 Institute for Research in Biomedicine (IRB Barcelona), Barcelona, Catalonia, Spain; Harvard Medical School, Howard Hughes Medical Institute, UNITED STATES

## Abstract

Clusters of differentiated cells contributing to organ structures retain the potential to re-enter the cell cycle and replace cells lost during development or upon damage. To do so, they must be designated spatially and respond to proper activation cues. Here we show that in the case of *Drosophila* differentiated larval tracheal cells, progenitor potential is conferred by the spatially restricted activity of the Snoo transcription cofactor. Furthermore, Dpp signalling regulated by endocrine hormonal cues provides the temporal trigger for their activation. Finally, we elucidate the genetic network elicited by Snoo and Dpp activity. These results illustrate a regulatory mechanism that translates intrinsic potential and extrinsic cues into the facultative stem cell features of differentiated progenitors.

## Author Summary

An important feature of organs is their ability to maintain their structure and function in spite of natural or accidental cell loss. This capacity is often sustained by so-called stem cells, which are able to provide new cells of the different types in the organ. In addition, some specialized cells, known as facultative stem cells, also retain the ability to re-enter the cell cycle and replace lost tissue. This process has to be very precisely regulated to provide for the maintenance of the tissues and organs while preventing uncontrolled cellular growth. We have analysed this mechanism in the *Drosophila* trachea; there, a group of Differentiated Adult Progenitor cells (or DAP cells) share the features of facultative stem cells as they remain quiescent during larval growth, reactivate their proliferation at the last larval stage and give rise to the different cell types of the adult tracheal network during metamorphosis. The DAP cells, conversely to the majority of the larval cells, do not enter endocycle and by doing so they acquire the features of adult progenitor cells. In this paper we identify the regulatory mechanism that integrates spatial and temporal cues to precisely activate the tracheal adult progenitor program.

## Introduction

Facultative stem cells have been defined as a particular class of differentiated cells that contribute to the structure and function of well-developed organs but remain multipotent; thus, upon damage due to either regular usage or injury they can proliferate and their progeny acquire the identities of different cell types that comprise the organ. While this property is fundamental to ensuring organ development and homeostasis, we still lack a detailed understanding of how these cells are set apart and how they express their progenitor features. We address this issue by the study of a group of progenitor cells in *Drosophila* with the features of facultative stem cells, namely the Differentiated Adult Progenitors (DAP) cells of the larval trachea [1].

Like most Drosophila larval cells, larval tracheal cells are polyploid and die at metamorphosis without contributing to the adult trachea [2]. However, among the larval tracheal cells, some cells escape the endocycle and by doing so acquire the features of progenitor cells of the adult trachea [3]. These cells remain quiescent during larval growth, reactivate their proliferation at the last larval stage and give rise to the different cell types of the adult tracheal network during metamorphosis [1, 4–6]. DAP cells belong to the dorsal trunks (DT), the main tracheal branches in the larvae and are specific to the second tracheal metamere (Tr2). The difference between the DT cells in Tr2 and those of the DT in other metameres is established by homeotic genes that exclude *fzr* expression from the DT cells in TR2 [3, 6]. *fzr* encodes the Drosophila homolog of the CDH1 protein of the E3 ubiquitin ligase APC/C and is the gene associated with endocycling in *Drosophila* [7–9].

While exclusion of *fzr* expression from the DT cells of TR2 sets a permissive state that allows for the deployment of the genetic program associated with the adult progenitor fate, it remains unclear what is the trigger for said progenitor program. Here we identify the Snoo transcriptional cofactor as the executor of the adult progenitor program, which is downregulated by Fzr in metameres other than TR2. However, while Snoo and Fzr are both spatially regulated, they do not show any temporal specificity and thus cannot account for the temporal control of DAP cell proliferation at the third larval stage (L3). In this regard, we show here that mitotic activation of DAP cells as well as expression of progenitor features also requires Decapentaplegic (Dpp)/Tgf-β signalling, which is not active in the DT cells at L2 but is activated at L3. Further, we show Dpp signalling to be under the positive control of Ecdysone and the negative control of Juvenile Hormone thus providing the readout for the transition from L2 to L3. Finally, we report that together, Snoo and Dpp regulate the mitotic factor String (Stg)/*cdc-25* as well as Broad(*br)*, a key regulator of metamorphosis to promote the proliferation and progenitor behaviour of DAP cells. In sum, we show how the spatial restriction of Snoo protein and timed activation of Dpp provides sufficient instruction for differentiated tracheal cells to fulfil their progenitor potential.

## Results and Discussion

**Fig 1:**
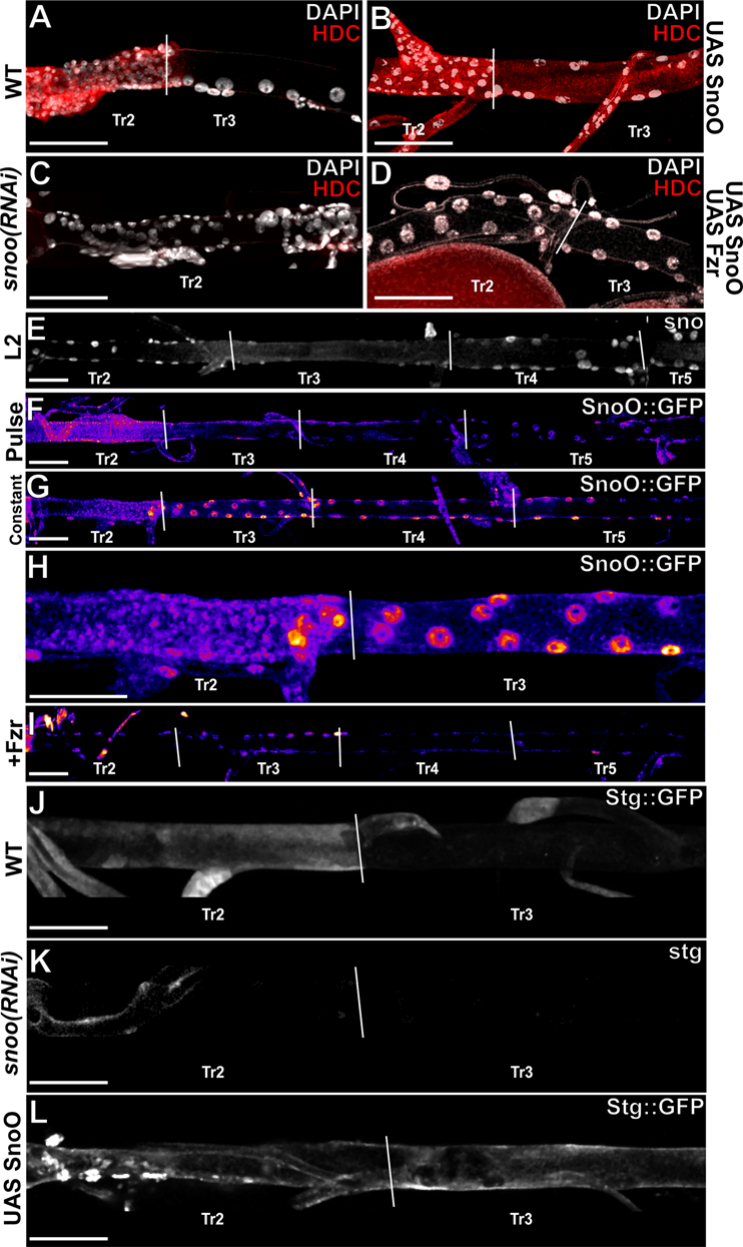
Snoo effects DAP cell behaviour downstream of Fzr. A)Wild type expression of Hdc. B)Ectopic expression of Hdc following forced tracheal over expression of *snoo* using *btl*-gal4 during L3. C)Loss of Hdc expression following RNAi mediated knockdown of *snoo* in the trachea via activation of *btl*-gal4 during L3. D)Expression of Hdc is lost when *snoo* is coexpressed with *fzr* in the trachea via *btl*-gal4 during L3. E)Expression of *snoo* as visualized by *snoo*-lacZ. F)Accumulation of Snoo::GFP following a pulse of tracheal expression (see methods). Red shows higher levels while blue/purple shows lower levels. G)Magnification of Tr2 and Tr3 showing nuclear accumulation of Snoo::GFP in DAP cells. H)Accumulation of Snoo::GFP when *btl*-gal4 is active continuously. I)Accumulation of Snoo::GFP when coexpressed with *fzr*. J)Wild type expression of Stg::GFP. K)Inactivation of *stg* expression after RNAi mediated knockdown of *snoo* in the trachea during L3. L)Ectopic Stg::GFP accumulation following *btl*-gal4 mediated tracheal overexpression of *snoo* during L3. Scale bars indicate 100um.

The genetic program associated with adult progenitor cell fate is suppressed by Fzr in the DT cells of metameres other than TR2 probably by downregulation of one or more specific transcription factors. Among the possible targets for Fzr mediated degradation, the mammalian Ski/Sno oncogenes seemed an interesting candidate [10] as they are both a target of CDH1 and a regulator of cell proliferation. We thus reasoned that the *Drosophila* homologue, Snoo, might play a role in DAP cell behaviour [10]. Like its mammalian counterparts, Snoo contains a highly conserved d-box motif, which likely results in targeting for degradation by the APC/C [11]. Consistent with the hypothesis that Snoo is responsible for activating the adult progenitor genetic program, forced overexpression of Snoo in the entire trachea results in ectopic expression of Headcase (Hdc), a marker for adult progenitor cell fate [3], whose expression in the wild-type is restricted to the DT cells in TR2 (Fig 1A and 1B) Conversely, RNAi mediated knockdown of Snoo causes loss of Hdc expression in the DT cells of TR2 (Fig 1C). Interestingly, some nuclei in the Tr2 DT of *snoo(RNAi)* trachea appear larger than their WT counterparts. This is likely due to an incomplete penetrance upon Snoo RNAi in the loss of *stg* expression, which is associated with the mitotic potential of the cells; thus these cells are likely larger due to the fact that they are at 4N after having gone through an S-phase and are paused at G2 [3, 6].

Transcription of the *snoo* gene occurs all over the DT larval tracheal cells including DAP cells as assessed by a construct driving lacZ expression under the control of the *snoo* promoter (Fig 1E)[12]. To examine whether Fzr promotes the degradation of the Snoo protein, and since we could not rely on any available antibody, and having failed in producing a Snoo specific antibody, we made use of a transgenic strain carrying a GFP tagged form of Snoo under the control of UAS promoter sequence [13]. Following a pulse of *btlGal4* mediated expression of the tagged form of Snoo (see mat and methods), we recovered tracheae that accumulated Snoo::GFP in the DT cells in the TR2 metamere, which do not express *fzr*. However, we observed very low levels of Snoo::GFP in the DT cells posterior to the TR2 metamere, which do express fzr (Fig 1F). Conversely, continuous expression of the same construct leads to Snoo::GFP accumulation along the DT cells of all metameres (Fig 1G and 1H) suggesting that under these circumstances endogenous Fzr protein cannot degrade the high amounts of induced Snoo::GFP protein, thus accounting for the general expression of adult progenitor markers (see above). Finally and consistent with these observations, accumulation of the DAP marker (Hdc) throughout the DT cells of all metameres upon forced overexpression of Snoo can be reversed by coexpression of Fzr (Fig 1D and 1I). All together these results identify Snoo as a transcription factor triggering the DAP genetic program and being antagonized by Fzr.

Upon tracheal overexpression of Snoo we also observe DT cell division (as indicated by pH3 staining) in the metameres where DT cells normally enter into endocycle and do not divide (S1A and S1B Fig). Since Fzr negatively regulates activity of the *stg* locus [3], thus preventing cell division, we reasoned that, similar to the case for Hdc, Snoo may also activate stg expression. In this way, Fzr mediated degradation of Snoo would account for the negative effect of Fzr on stg expression. Consistent with this hypothesis, *stg* expression in the DT cells in Tr2 is lost following RNAi-mediated knockdown of Snoo (Fig 1J and 1K) while Stg-GFP accumulates ectopically in the DT cells posterior to the TR2 metamere upon overexpression of *snoo* (Fig 1L). Thus, Snoo appears to be necessary and sufficient to trigger both proliferation and expression of adult progenitor cell markers in the DT cells.

**Fig 2:**
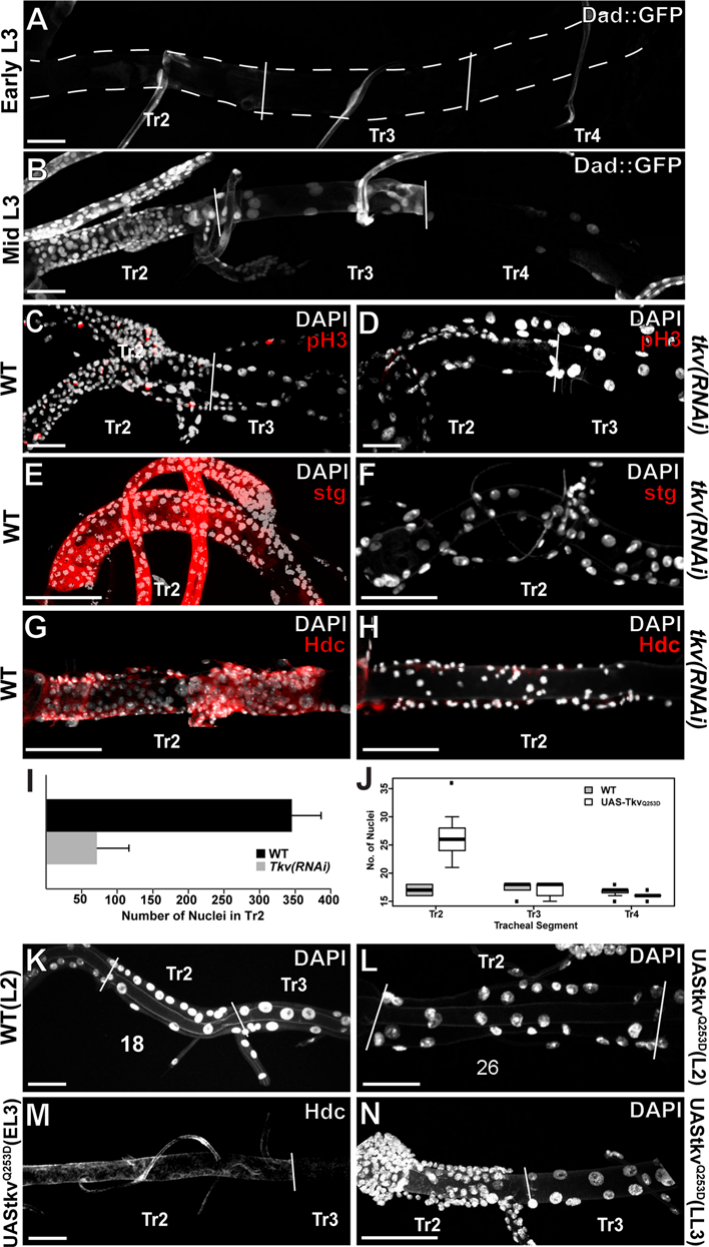
Dpp signalling temporally activates DAP cells. A)Expression of Dpp signalling marker Dad::GFP in early L3. B)Expression of Dad::GFP in mid L3. C)Wild type cells marked with mitotic marker pH3 showing division in DAP cells. D)Loss of mitotic potential as shown by pH3 following RNAi mediated knockdown of *tkv* in the trachea via *btl*-gal4/UAS-*tkv*RNAi during L3. E)Wilde type expression of *stg*. F)Inactivation of *stg* expression following RNAi mediated knockdown of *tkv* in the trachea via *btl*-gal4/UAS-*tkv*RNAi during L3. G)Wild type expression of Hdc in DAP cells. H)Loss of Hdc expression in DAP cells following RNAi mediated knockdown of *tkv* in the trachea via *btl*-gal4/UAS-*tkv*RNAi during L3. I)Average number of nuclei found in Tr2 DT of WT and *tkv(RNAi)* tracheae. Error bars are standard deviation from the mean. T-test p < .0001. J)Number of nuclei found in Tr2-Tr4 of WT and UAS-*tkv*^*Q154D*^ trachea at the L2-L3 molt. K)Number of DAP cells found in trachea of WT molting L2 larvae. L)Extra cells observed in Tr2 of molting L2 larvae following forced expression of constitutively active *tkv*^*Q154D*^ via *btl*-gal4/UAS-*tkv*^*Q154D*^ starting at L1. M)Early expression of Hdc in early L3 trachea following forced expression of *tkv*^*Q154D*^ via *btl*-gal4/UAS-*tkv*^*Q154D*^ starting at L1. N)Lack of ectopic mitosis outside Tr2 in L3 trachea following forced expression of *tkv*^*Q154D*^ via *btl*-gal4/UAS-*tkv*^*Q154D*^ during L3. Scale bars indicate 100um.

Expression of specific markers and onset of proliferation of DAP cells occurs at mid third larval instar (L3), the last larval stage before pupariation and metamorphosis. Thus, because *snoo* is transcribed at earlier larval stages (Fig 1E) *snoo* expression cannot be responsible for providing the temporal cue for L3 activation of the DAP cell program, suggesting that a temporally regulated trigger must cooperate with Snoo. To investigate which this temporally regulated trigger might be we took into consideration previous reports on the interaction between Ski/Sno proteins and TGF-beta signalling [12–14]. Thus, we decided to investigate the role of the Dpp pathway, the *Drosophila* homolog of TGF-beta, in DAP cell proliferation. First, we analysed the timing of Dpp pathway activity by the use of a GFP tagged version of Daughters against dpp (Dad), a reporter of Dpp signalling *[15]*. At Early L3, we do not observe Dad::GFP expression in the DT cells (Fig 2A), where it is only detected by mid L3 (Fig 2B), the time of mitotic activation of DAP cells [3]. This pattern mimics that of the phosphorylated form of the R-SMAD, Mothers against dpp (Mad) (S2A–S2C Fig). It should be noted that while activation of Dpp signalling during L3 in the wild-type trachea initiates at the anterior metameres it eventually occurs along the DT cells of all the metameres. Furthermore, we find the mitotic potential of DAP cells to be impaired following RNAi mediated knockdown of the DPP receptor Thickveins (Tkv) as evidence by loss mitotic marker pH3 (Fig 2C, 2D and 2I) as well as loss of expression of stg (Fig 2E and 2F). The abrogation of mitosis via Tkv knockdown is consistent with the recently reported role of Dpp signalling in promoting remodelling in the larval trachea [16]. Remarkably, Dpp signalling is not only required to promote cell proliferation but also for the DAP genetic program as we also observe loss of Hdc expression following RNAi mediated knockdown of Tkv (Fig 2G and 2H) and Mad (S2H Fig). Consistent with the Dpp pathway providing a temporal cue for DAP cell proliferation, we observe extra Tr2 DT cells in molting L2 larvae upon precocious Dpp signalling by means of the constitutively activated receptor encoded by the *tkv*^*Q253D*^ allele [17] (Fig 2J, 2K and 2L). Precocious activation of the Dpp pathway also triggers an earlier activation of Hdc as well (Figs 2M, S2D–S2G). As Dpp signalling by L3 occurs along the DT cells of all the metameres it seems therefore not to be responsible for the spatial restriction of the DAP cell genetic program to the DT cells of Tr2. Consistently, ubiquitous tracheal expression of the allele encoding the constitutively activated receptor tkv^*Q253D*^ does not induce either cell division or ectopic *hdc* expression outside the Tr2 metamere (Fig 2J, 2M and 2N). All together, these results indicate that Dpp signalling provides the temporal trigger for the activation of the adult progenitor cell markers and for their proliferation.

**Fig 3:**
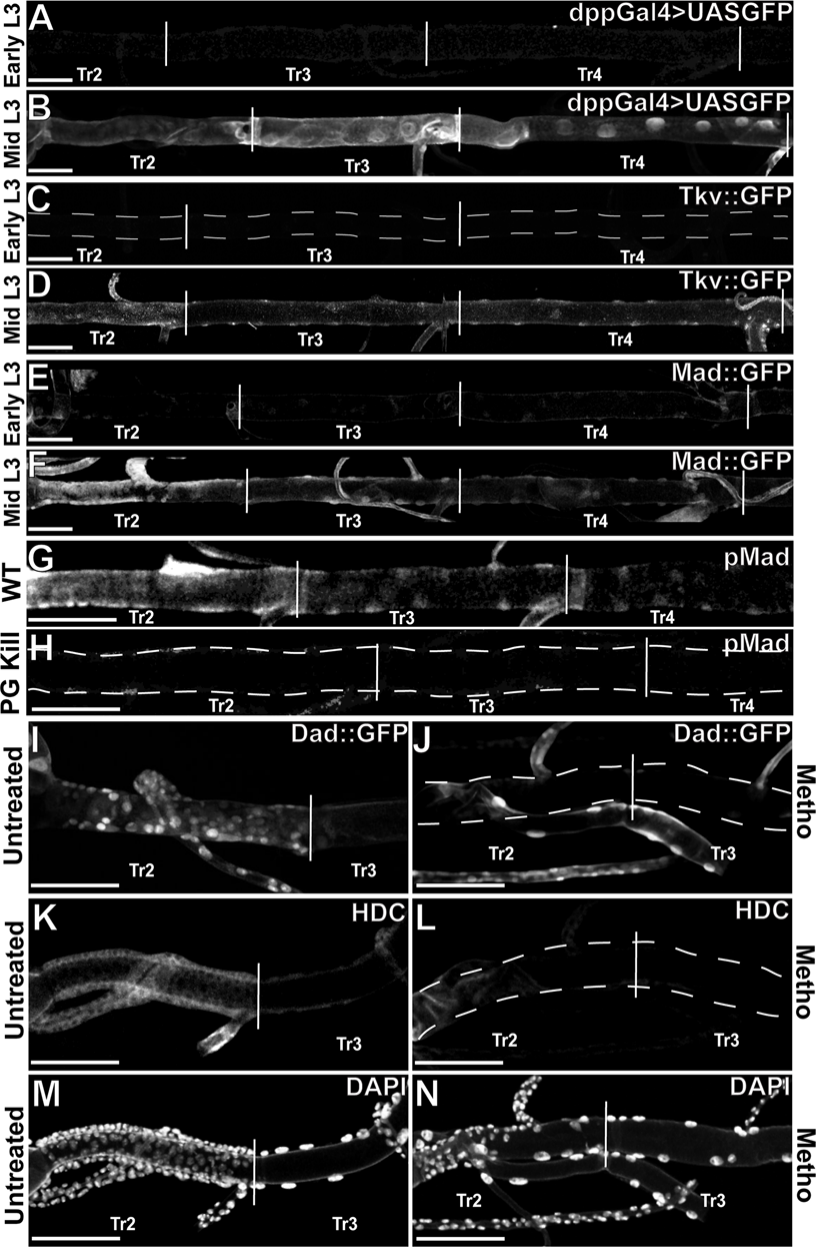
Dpp signalling is under temporal control of Ecdysone. A)Expression of *dpp*-gal4<UAS-gfp in early L3 trachea. B)Expression of dpp-gal4<UAS-gfp at mid L3. C)Expression of Tkv::GFP in early L3 trachea. D)Expression of Tkv::GFP in mid L3 trachea. E)Expression of Mad::GFP in early L3 trachea. F)Expression of Mad::GFP in mid L3 trachea. G)Wild type activation of dpp as shown by pMad. H)Loss of pMad accumulation after genetic ablation of Prothoracic Gland. I)Wild type expression of Dad::GFP in DAP cells. J)Loss of Dad::GFP in DAP cells following treatment with Methoprene. k)Wild type expression of Hdc. L) Wild type nuclei showing normal mitotic potential. M)Loss of Hdc expression in DAP cells following treatment with Methoprene. N)Decreased mitotic potential in DAP cells following treatment with Methoprene. Scale bars indicate 100um.

We then addressed how Dpp signalling might be so timely regulated and found that expression of Dpp itself as inferred via dpp-gal4<UAS-gfp [18, 19] (Fig 3A and 3B, S3A–S3F Fig), the Dpp receptor Tkv as shown by GFP tagged Tkv protein [20] (Fig 3C and 3D) and the downstream activator Mad as shown by GFP tagged Mad protein [21] (Fig 3E and 3F) in the DT cells is turned on at mid L3. As timely activation of DAP cells requires hormonal input via Ecdysone [3], it is likely that Dpp and Tkv expression is itself dependent upon ecdysone signalling. Indeed, genetic ablation of the Ecdysone producing Prothoracic Gland (material and methods and [3] during the third larval instar abrogates the activation of the dpp pathway (Fig 3G and 3H). However there are several cycles of ecdysone activation during larval life, with ecdysone levels peaking at each molt, whereas Dpp signalling is active in the DT cells only after the molt from L2 to L3, suggesting the activity of another temporal regulatory input. We reasoned such an additional input could be the Juvenile Hormone (JH). Levels of JH stay at high levels until the third larval stage and then drop to very low levels [22]. In the cockroach *Blatella*, this drop in JH levels is required for metamorphosis to be triggered by a peak of Ecdysone [23]. Similarly, Dpp, Mad and Tkv expression in the DT cells might be under the positive control of ecdysone and the negative control of JH. Due to complexity of JH signaling, it was not feasible to genetically manipulate the JH pathway [24]. We instead chose to prevent the reduction in JH signalling by exposing larvae to the JH agonist methoprene [25]. Consistent with JH regulation of Dpp signalling, we observed not only reduced signalling activity of DPP, as seen by the Dad::GFP reporter, but also a reduction of cell division as well as loss of Hdc expression in Tr2 DT DAP cells (Fig 3I–3N).

**Fig 4:**
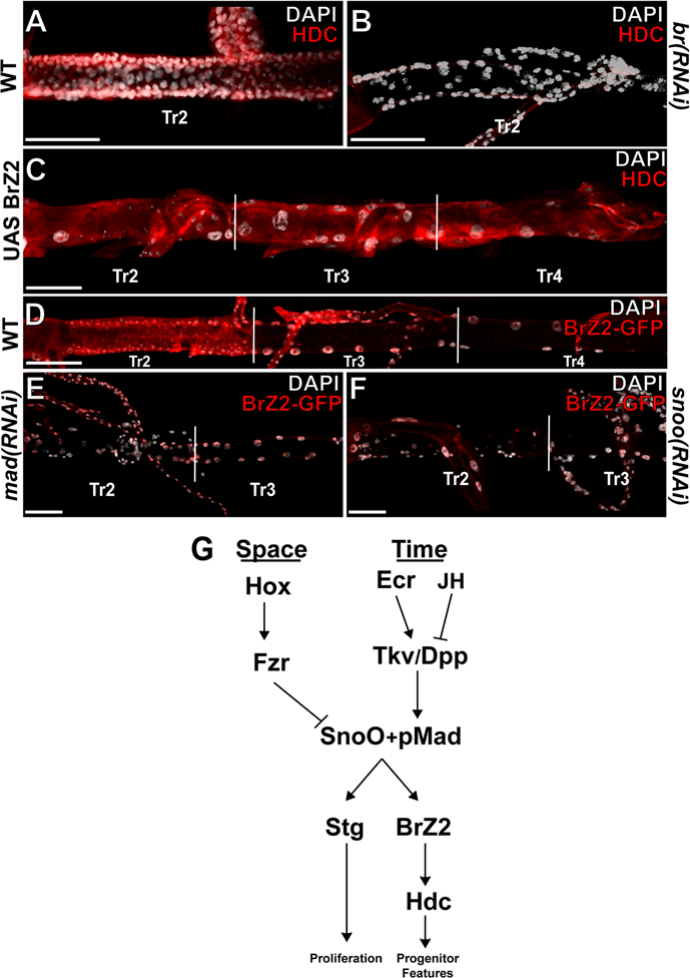
Dpp/Snoo activate the DAP genetic program via BrZ2. A)Wild type expression of Hdc in DAP cells. B)Loss of Hdc expression in DAP cells following RNAi mediate knockdown of *br* via *btl*-gal4/UAS-*br*RNAi activation in L3. C)Ectopic expression of Hdc following forced tracheal expression of bBrZ2 via *btl*-gal4/UAS-*brZ2* during L3. D)Wild type expression of BrZ2 in L3 trachea. E)Loss of BrZ2::GFP expression in DAP cells following RNAi mediated knockdown of *mad* in the trachea via *btl*-gal4/UAS-*mad*RNAi during L3. F)Loss of BrZ2::GFP expression in DAP cells following RNAi mediated knockdown of *snoo* in the trachea via *btl*-gal4/UAS-*snoo*RNAi during L3. G)Model of Spatio-temporal regulation of DAP cell potential and activation. Snoo activity is spatially restricted to DAP cells via Hox mediated activation of Fzr. Dpp pathway activation by Ecdysone in the absence of Juvenile Hormone acts as the temporal trigger for DAP cell activation. Snoo and pMad activate *stg* expression, initiation mitotic growth of DAP cells as well as BrZ2 which activates *hdc*. Scale bars indicate 100um.

While the above results show that Dpp signalling is required for *hdc* expression (Fig 2H), an analysis with the matscan application [26] did not predict Mad binding sites in the *hdc* promoter region, suggesting that *hdc* might not be a direct target of the Dpp pathway. Instead, our predictions showed several binding sites for Mad in the *br* locus and for Br in the *hdc* locus. The *br* gene is a well-known target of ecdysone signalling [27] and is activated in the DT cells of Tr2 at L3 [3]. We thus examined 1) whether activation of *hdc* expression by Dpp signalling might be mediated by *br*. and 2) whether activation of *br* expression by ecdysone might be mediated by Dpp signalling. Consistent with *hdc* expression being dependent on Br function, RNAi mediated knockdown of Br results in the loss of Hdc in the DT cells of the TR2 metamere (Fig 4A and 4B). To further analyse the role of br in hdc regulation, we explored whether br activity was sufficient to activate *hdc* expression in all the DT cells. *br* encodes four isoforms (Z1-Z4) required for various cellular processes during metamorphosis [28, 29]. We forced the expression of each of distinct isoform and found that the Z2 isoform of Br was sufficient to activate *hdc* in the rest of the trachea (Fig 4C). Altogether, these results place *hdc* expression downstream of *br*. Finally, and consistent with *br* expression being a target for Dpp signalling, RNAi mediated knockdown of Mad resulted in loss of *br*-Z2 expression in DAP cells (Fig 4D and 4E). In addition, we find the expression of the *br*-Z2 isoform in the DT cells of TR2 to be under the control of Snoo as this accumulation is lost upon RNAi mediated knockdown of Snoo (Fig 4D and 4F). These results indicate that, as is also the case for *stg* expression, *br* expression integrates the spatial and temporal triggers from Fzr/Snoo and ecdysone/Dpp signalling respectively.

Interestingly, RNAi mediated knockdown of Br did not affect either DT cell division or *stg* expression (S4A and S4B Fig), which suggests a split in the activation of the programs leading to DAP cells specification and proliferation downstream of Snoo/Dpp.

Finally, we want to note that the requirement of Mad for *br* and *stg* expression does not imply that the Dpp pathway solely mediates all the effect of ecdysone in these genes. In this regard, we observed overlap at the promoter regions of *br* and *stg* when comparing ChIP-Seq data of EcR binding produced from whole animals at prepupal stages with predicted Mad binding sites using the Gbrowse function of modENCODE [30]. These observations suggest the combined activity of Ecdysone receptor as well as Mad/Snoo might ensure a robust activation of the DAP cell genetic program.

The main distinction between undifferentiated progenitors and “facultative stem cells” is the continuous growth required for normal cellular turnover of the first vs activation of the later in singular conditions such as injury [31, 32]. Thus, facultative stem cells which functionally contribute to an organ, yet retain their multipotency, must ignore the signals that mediate the continual cellular turnover that maintains tissue homeostasis. Only upon specific events should they activate and realize their potential as progenitors. In the fly, continuous growth of undifferentiated progenitors stimulated by hormonal signalling is exemplified by the growth of the imaginal discs throughout larval life. On the other hand, the precise triggering of facultative stem cell growth can be exemplified by the activation of tracheal DAP cells. Where the growth of imaginal disc cells is continuous, activation of DAP cells requires additional distinct spatial and temporal cues. The findings here reported show how spatial restriction of potential via Hox mediated activation of Fzr and degradation of Snoo ensures that the temporal cue of Ecdysone in the absence of the juvenile hormone and mediated by activation of Dpp signalling will be interpreted by the correct population of cells in response to the correct organismal context (Fig 4G). These results illustrate a mechanism by which factors acting in differentiated cells to maintain the potential for pluripotency cooperate with the factors triggering their transition from quiescence to proliferation in order to carry out their function in regenerative growth; this may provide a general framework for studying facultative stem cells and lend insight into what may be missing in similar cells that are incapable of such potential and/or to respond to the temporal trigger to activate their potential.

## Materials and Methods

### *Drosophila* stocks and genetics

The following flies were obtained from the Bloomington Stock Center: *UAS-tkvRNAi*, *UAS-snooRNAi*, BrZ2::GFP, Mad::GFP, *UAS-brZ1*, *UAS-brZ2*, *UAS-brZ3*, *and UAS-brZ4*. *UAS-madRNAi* and *UAS-brRNAi*, were obtained from the VDRC. Stg::GFP (YD0246) was obtained from the Yale Flytrap Project. The following strains were given by: *UAS-snoo; UAS-fzr* (Rosa Barrio), *UAS-snoo* (Rosa Barrio), *UAS-snoo*::*GFP* (Jose de Celis), the R6.4 *stg-lacZ* (Lehman et al, 1999), *UAS-tkv*^*Q253D*^ (Lecuit et al 1996), Tkv::GFP (Hsiung et al, 2005), *dpp-Gal4<UAS-GFP* (María Domínguez), *snoo-lacZ*(SH1402) (Stuart Newfeld) and Dad::n::GFP (Enrique Martin-Blanco).

### Immunochemistry

Larval tracheae were dissected at either L2 or L3 larval instar and immunostained according to standard protocols. The following primary antibodies were used: mouse anti-Hdc (1:1), mouse anti-β-galactosidase 40.1a (1:200) from the Hybridoma Bank, rabbit anti-PH3 (Ser) (1:100) from Cell signaling, rabbit anti pMad (1:100) a gift from Gines Morata and goat anti-GFP (1:500) from Abcam. Secondary antibodies labeled with Alexa 488, Alexa 555, or Alexa683 were obtained from Molecular Probes. Micrographs were acquired with Leica SP5 and SPE confocal microscopes and images were processed with FIJI.

### *snoo* expression

In order to assess transcriptional activation of *snoo*, we utilized the SH1402 lacW insertion in the *snoo* locus. Tracheae from L2 and L3 larvae were assayed for LacZ expression.

### Snoo knockdown and overexpression

In order to knockdown Snoo, virgin *UAS-snooRNAi* flies were crossed with *btl-Gal4<UAS-gfp;tub-Gal80* males. In order to overexpress Snoo gene throughout the trachea, virgin *UAS-snoo* flies were crossed with *btl-Gal4<UAS-gfp;tub-Gal80* males. The resulting progeny were reared at the permissive temperature for GAL80 (18°C) until L2, when they were shifted to the non-permissive temperature for GAL80 (29°C) and maintained until late L3 and assayed for cell division and Hdc expression. In or der to coexpress Snoo and Fzr, virgin *Uas-snoo;Uas-fzr* flies were crossed with *btl-Gal4<UAS-gfp;tub-Gal80* males. The resulting progeny were reared at the permissive temperature for Gal80 (18°C) until L2, when they were shifted to the non-permissive temperature for Gal80 (29°C) and maintained until late L3 and assayed for cell division and marker expression.

### Snoo::GFP pulse expression

In order to assess the accumulation of Snoo protein in the presence of Fzr, virgin *UAS-snoo*::*GFP* flies were crossed with *btl-Gal4;tub-Gal80* males. In order to continuously express Snoo::GFP larvae were reared at the non-permissive temperature for Gal80 (29°C) until late L3 and then assayed for GFP expression. For the pulsed expression of Snoo::GFP, larvae were reared at the permissive temperature for Gal-80 (18°C) until late L2, shifted to the non-permissive temperature for Gal80 (29°C) for one day and then shifted back down to the permissive temperature for Gal80 (18°C) until late L3 and assayed for GFP expression. In order to observe the effect of coexpression of Snoo::GFP with Fzr, *UAS-snoo*::*GFP* flies were crossed with *UAS-fzr* flies. The resulting *UAS-Snoo;UAS-fzr* males were crossed with virgin *btl-Gal4;tub-Gal80* flies. The resulting progeny were reared at the permissive temperature for Gal80 (18°C) until L2, when they were shifted to the non-permissive temperature for Gal80 (29°C) and maintained until late L3 and assayed for cell division and GFP expression.

### Snoo regulation of *stg*

In order to assess the regulation of *stg* via Snoo, *UAS-snoo* flies were crossed with Stg::GFP flies and *UAS-snooRNAi* flies were crossed with *stg-lacZ* flies. *UAS-snoo;*Stg::GFP males were crossed with virgin *btl-Gal4;tub-Gal80* flies. The resulting progeny were reared at the permissive temperature for Gal80 (18°C) until L2, when they were shifted to the non-permissive temperature for Gal80 (29°C) and maintained until late L3 and assayed for cell division and GFP expression. *stg-lacZ; UAS-snooRNAi* males were crossed with virgin *btl-Gal4<UAS-gfp;tub-Gal80* flies. The resulting progeny were reared at the permissive temperature for Gal80 (18°C) until L2, when they were shifted to the non-permissive temperature for Gal80 (29°C) and maintained until late L3 and assayed for cell division and LacZ expression.

### Dpp pathway activation and component expression

In order to assess activation of the Dpp pathway in DAP cells as well as expression of Dpp pathway components, tracheae from Dad::n::GFP, dpp-Gal4<UAS-GFP, Tkv::GFP and Mad::GFP were taken from L2 and various stages of L3 larvae and assayed for GFP expression. Wild-type tracheae were also stained for phosphorylated Mad (pMad) during L3.

### Dpp regulation of DAP cell division and progenitor features

In order to assess the role of Dpp signalling in DAP cell proliferation and progenitor behaviour, virgin UAS-tkvRNAi and UAS-madRNAi flies were crossed with btl-Gal4<UAS-gfp;tub-Gal80 males. The resulting progeny were reared at the permissive temperature for GAL80 (18°C) until L2, when they were shifted to the non-permissive temperature for GAL80 (29°C) and maintained until late L3 and assayed for cell division and Hdc expression. In order to assess the regulation of stg via Tkv, UAS-tkvRNAi flies were crossed with stg-lacZ flies. stg-lacZ;UAS-tkvRNAi males were crossed with virgin btl-Gal4<UAS-gfp;tub-Gal80 flies. The resulting progeny were reared at the permissive temperature for Gal80 (18°C) until L2, when they were shifted to the non-permissive temperature for Gal80 (29°C) and maintained until late L3 and assayed for cell division and LacZ expression. In order to precociously activate the Dpp pathway in L2 larvae, virgin UAS-tkvQ253D flies were crossed with btl-Gal4<UAS-gfp;tub-Gal80 males. The resulting progeny were reared at the permissive temperature for GAL80 (18°C) until L1, when they were shifted to the non-permissive temperature for GAL80 (29°C) and maintained until late molting L2, early L3 and late L3and assayed for cell division and Hdc expression.

### Prothoracic Gland ablation

In order to assess dpp pathway activation in the absence of the Prothoracic Gland, a GAL80 suppressible *phantom-gal-4* (P0206-GAL-4) was used to drive Reaper expression in the Prothoracic Gland during the 2^nd^ larval instar. Virgin *UAS-rpr;tub-gal80* flies were mated with *P0206-Gal4* males. Larvae were maintained at the permissive temperature for GAL80 (18°C) until the 2^nd^ larval instar when they were shifted to the non-permissive temperature (29°C) and maintained there for at least two days at L3 before being assayed for phosphorylated Mad.

### Regulation of Dpp pathway by Juvenile Hormone (JH)

In order to assess the effect of prolonged JH signaling on Dpp pathway activity as well as DAP cell proliferation and progenitor features, Dad::n::GFP larvae were treated with methoprene [25] (21.1ng/ul) applied topically once daily starting at L2 until late L3and assayed for GFP expression, cell division and Hdc expression.

### Br regulation of progenitor features

In order to test the necessity and sufficiency of Br proteins for the expression of Hdc, virgin UAS-brRNAi, UAS-brZ1, UAS-brZ2, UAS-brZ3. UAS-brZ4 flies were crossed with crossed with btl-Gal4<UAS-gfp;tub-Gal80 males. The resulting progeny were reared at the permissive temperature for GAL80 (18°C) until L2, when they were shifted to the non-permissive temperature for GAL80 (29°C) and maintained until late L3 and assayed for cell division and Hdc expression. In order to test the regulation of br-Z2 by Mad and Snoo, Br-Z2::GFP flies were crossed with UAS-snooRNAi and UAS-madRNAi flies. The resulting UAS-snooRNAi/Br-Z2::GFP and UAS-madRNAi;Br-Z2::GFP males were crossed with virgin btl-Gal4;tub-Gal80 flies. The resulting progeny were reared at the permissive temperature for Gal80 (18°C) until L2, when they were shifted to the non-permissive temperature for Gal80 (29°C) and maintained until late L3 and assayed for cell division and GFP expression.

### Transcription factor binding site predictions

The matScan software [26] was used to scan promoter regions of 2000 nucleotides upstream of the genes of interest for possible binding sites for known transcription factors. Results were filtered by the default value of the matScan software (hits above 80% of the maximum possible score value). Hits were also filtered according to mean conservation scores, requiring a minimum score of 0.9. To further refine the candidate hits, we performed a permutation test by randomly selecting 500 regions of 2000 nucleotides from the dm3 genome and repeating the analysis for all PWMs. We used a cutoff of 0.05 for the resulting p-values. Genomic coordinates were obtained by the biomaRt package of Bioconductor [33] with the dm3 version of the Drosophila melanogaster genome. We used 652 position weight matrices (PWM) from the JASPAR database [34] downloaded from (http://pgfe.umassmed.edu/ffs/DownloadPWM.php?Type=FM&&IsPublic=1). We used the evolutionary conservation track from UCSC [35] for 14 species close to Drosophila melanogaster.

## Supporting Information

S1 Fig Ectopic cell division in following forced expression of Snoo. A) Extranumerary diploid cells observed in Tr3-5. Arrowheads show small, diploid nuclei resulting from ectopic mitosis. B) Mitotic cells in DT of Tr3-5 visualized by mitotic marker pH3 as well as extranumerary diploid cells (arrowheads). Scale bars represent 100um.
(TIF)

S2 Fig Activation of Dpp pathway, Hdc expression dynamics and regulation of progenitor features by Mad. A-C) Activation of Dpp pathway throughout the trachea visualized by pMad. D-G) Expression of Hdc in DAP cells starting from L2 (B) through late L3 (E). F) Loss of mitotic potential and expression of Hdc following RNAi mediated knockdown of Mad. Scale bars represent 100um.
(TIF)

S3 Fig Expression of Dpp in DT through L3. A-F) Expression of Dpp visualized via *dpp-gal4<UAS-gfp* from early L3 (A) through late L3 (F). Scale bars represent 100um
(TIF)

S4 Fig Proliferation potential of DAP cells after br RNAi. A) Bar graph showing average number of nuclei in the DT of Tr2 of wild type and *br(RNAi)* trachea. Error bars represent standard deviation from the mean. T-test p = .88. B) Wild type expression of Stg::GFP in *br(RNAi)* trachea. Scale bar represents 100um.
(TIF)
