# Supplementary figures and images for: Snoo and Dpp Act as Spatial and Temporal Regulators Respectively of Adult Progenitor Cells in the Drosophila Trachea
Source: PLoS Genet. 2016 Mar 4;12(3):e1005909. doi: 10.1371/journal.pgen.1005909 (PMC4778947; doi:10.1371/journal.pgen.1005909)

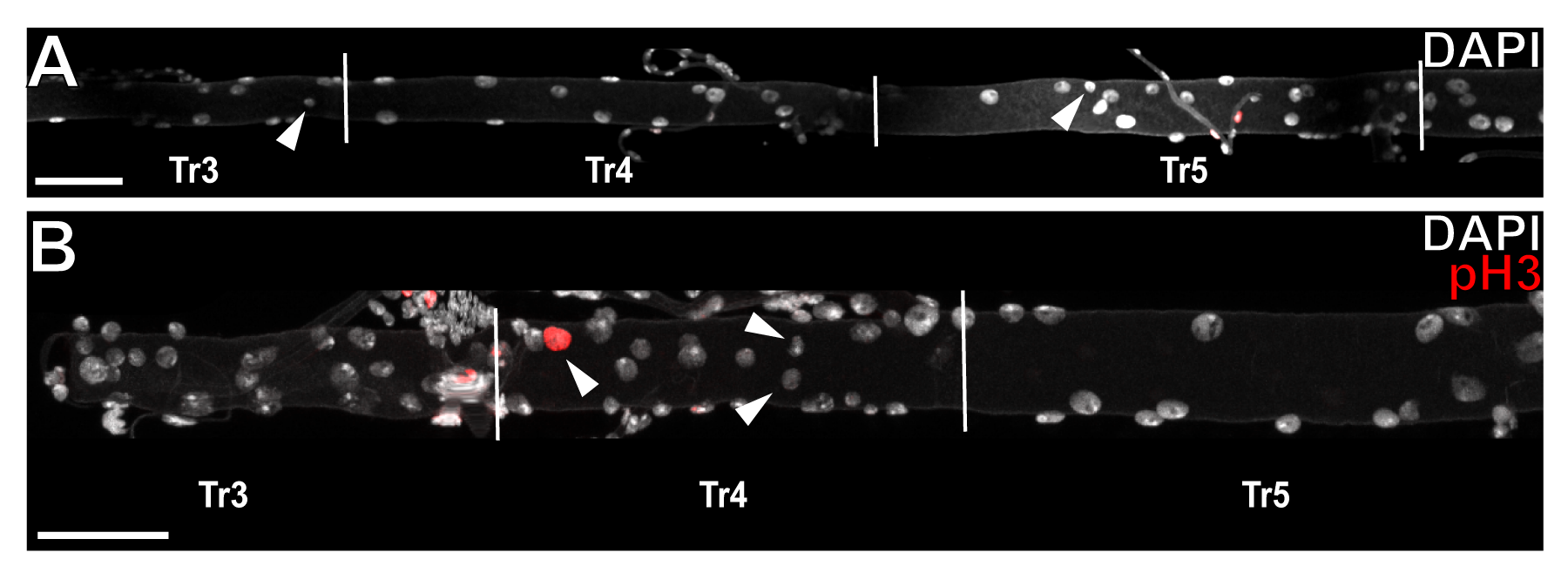

Supplement: S1 Fig — A) Extranumerary diploid cells observed in Tr3-5. Arrowheads show small, diploid nuclei resulting from ectopic mitosis. B) Mitotic cells in DT of Tr3-5 visualized by mitotic marker pH3 as well as extranumerary diploid cells (arrowheads). Scale bars represent 100um. (TIF) [file pgen.1005909.s001.tif]

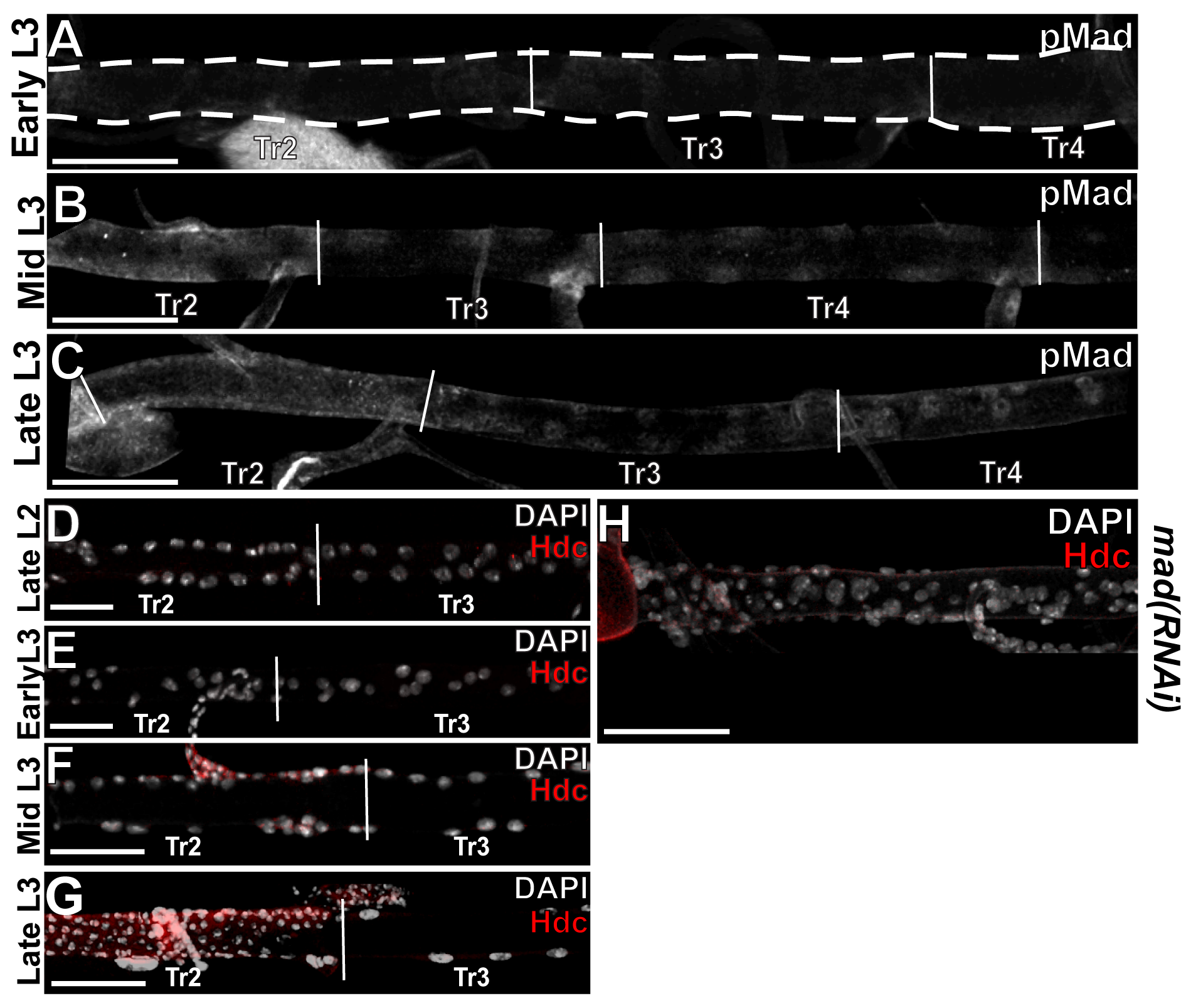

Supplement: S2 Fig — A-C) Activation of Dpp pathway throughout the trachea visualized by pMad. D-G) Expression of Hdc in DAP cells starting from L2 (B) through late L3 (E). F) Loss of mitotic potential and expression of Hdc following RNAi mediated knockdown of Mad. Scale bars represent 100um. (TIF) [file pgen.1005909.s002.tif]

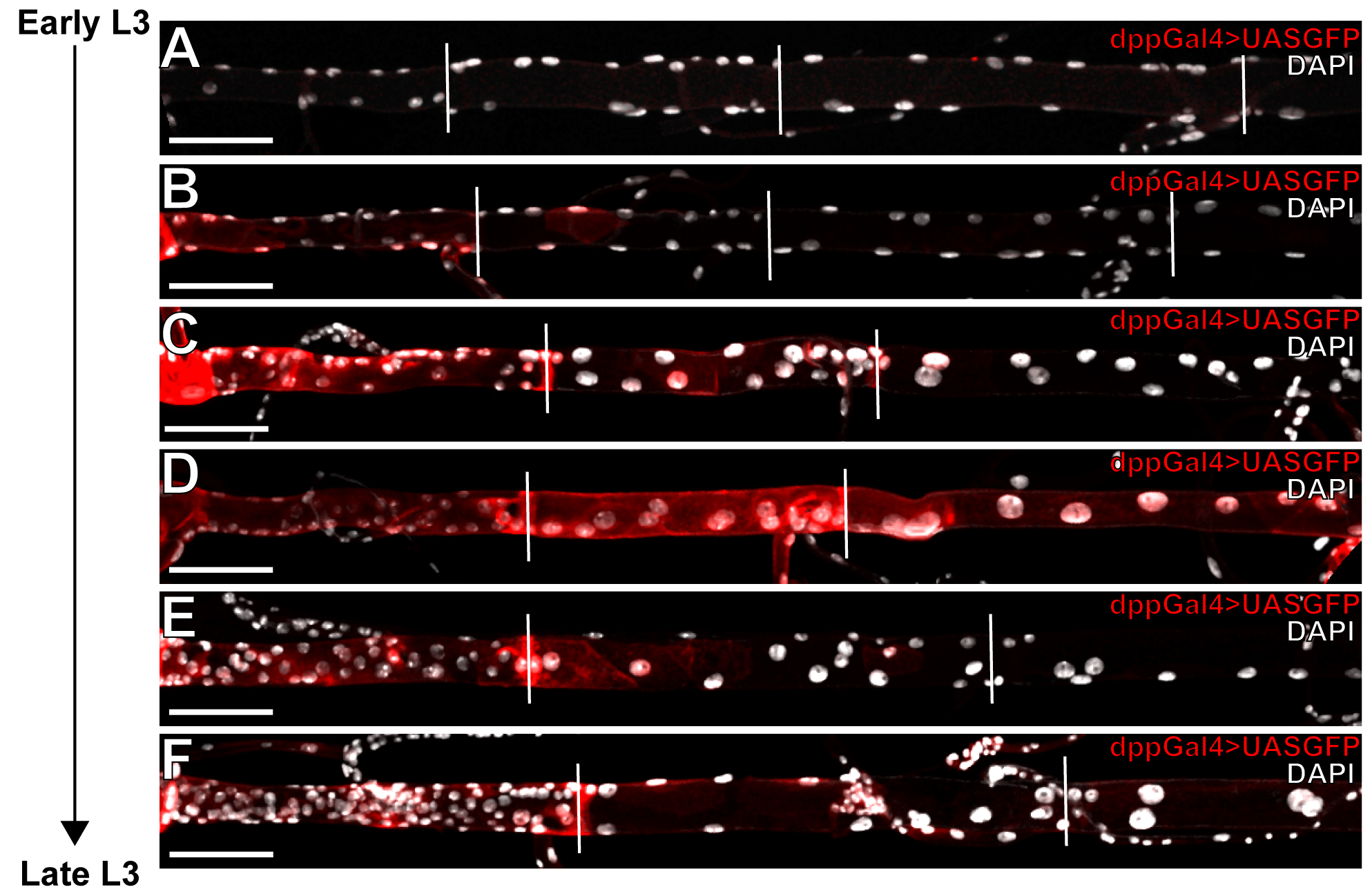

Supplement: S3 Fig — A-F) Expression of Dpp visualized via dpp-gal4<UAS-gfp from early L3 (A) through late L3 (F). Scale bars represent 100um (TIF) [file pgen.1005909.s003.tif]

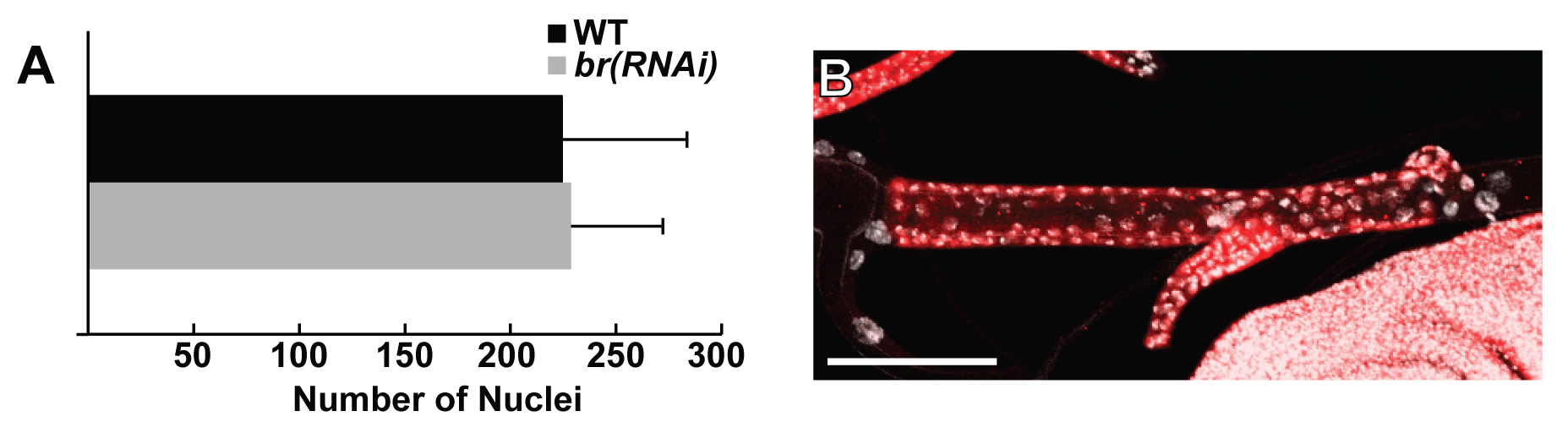

Supplement: S4 Fig — A) Bar graph showing average number of nuclei in the DT of Tr2 of wild type and br(RNAi) trachea. Error bars represent standard deviation from the mean. T-test p = .88. B) Wild type expression of Stg::GFP in br(RNAi) trachea. Scale bar represents 100um. (TIF) [file pgen.1005909.s004.tif]
